# 

**Published:** 1878-01

**Authors:** 


### Table of Contents.

### ORIGINAL COMMUNICATIONS.

Successful Operation for Strangulated Hernia in a Young Lady. *By Henry F. Andrews, M.D., Washington, Ga.* .......... 577

Hemorrhage from the Bowels. *By E. F. Starr, M.D., Nacoochee, Ga.* 581

Botany in its Relations with Medicine. *By W. T. Grant, M.D., Atlanta, Ga.* .......... 585

Atlanta Medical College Clinic. *Services of J. G. Westmoreland, M. D.* 590

Abnormal Condition of the Digestive Organs. *By Fred. King, M. D., Atlanta, Ga.* .......... 593

### REPORTS OF SOCIETIES.

Atlanta Academy of Medicine .......... 597

### SELECTIONS.

Case of Acute Tetanus following a Wound in the Foot .......... 605
Some Cases of Arm Presentation .......... 607
Electro-Physiology .......... 609

### EDITORIAL.

Illegitimate Advertising .......... 627
To Patrons .......... 628
Medical Society of the District of Columbia .......... 628

### BIBLIOGRAPHICAL.

Guide to Therapeutics and Materia Medica .......... 630
The Action of Medicines .......... 631
The Elements of Therapeutics .......... 632
Outlines of Modern Chemistry, etc .......... 633
The Ear—its Anatomy, Physiology and Diseases .......... 633
How to use the Ophthalmoscope .......... 634
Editorial Correspondence .......... 634, 635

### EXCERPTA.

Excipients .......... 637
Treatment of Diphtheria .......... 638
Orthopœdic Treatment of Paralysis of the Ocular Muscles .......... 639
Indications and Contra-Indications for Atropine Calabar in Eye Practice .......... 639
Local Effect of Quinine in Diphtheria .......... 640
Curari-Woorari—a cure for Hydrophobia .......... 640

